# RNA helicase DDX3 maintains lipid homeostasis through upregulation of the microsomal triglyceride transfer protein by interacting with HNF4 and SHP

**DOI:** 10.1038/srep41452

**Published:** 2017-01-27

**Authors:** Tsung-Yuan Tsai, Wei-Ting Wang, Hao-Kang Li, Wei-Ju Chen, Yu-Hong Tsai, Chi-Hong Chao, Yan-Hwa Wu Lee

**Affiliations:** 1 Department of Biological Science and Technology, National Chiao-Tung University, Hsinchu 300, Taiwan; 2 Institute of Biochemistry and Molecular Biology, National Yang-Ming University, Taipei 112, Taiwan

## Abstract

Multifunctional RNA helicase DDX3 participates in HCV infection, one of the major causes of hepatic steatosis. Here, we investigated the role of DDX3 in hepatic lipid metabolism. We found that HCV infection severely reduced DDX3 expression. Analysis of intracellular triglyceride and secreted ApoB indicated that lipid accumulations were increased while ApoB secretion were decreased in DDX3 knockdown HuH7 and HepG2 cell lines. Down-regulation of DDX3 significantly decreased protein and transcript expression of microsomal triglyceride transfer protein (MTP), a key regulator of liver lipid homeostasis. Moreover, DDX3 interacted with hepatocyte nuclear factor 4 (HNF4) and small heterodimer partner (SHP), and synergistically up-regulated HNF4-mediated transactivation of *MTP* promoter via its ATPase activity. Further investigation revealed that DDX3 interacted with CBP/p300 and increased the promoter binding affinity of HNF4 by enhancing HNF4 acetylation. Additionally, DDX3 partially relieved the SHP-mediated suppression on *MTP* promoter by competing with SHP for HNF4 binding which disrupted the inactive HNF4/SHP heterodimer while promoted the formation of the active HNF4 homodimer. Collectively, these results imply that DDX3 regulates *MTP* gene expression and lipid homeostasis through interplay with HNF4 and SHP, which may also reveal a novel mechanism of HCV-induced steatosis.

Hepatic steatosis, characterized by lipid accumulation in hepatocyte cytoplasm, is the initial step of nonalcoholic steatohepatitis, liver fibrosis and liver cirrhosis which could then lead to hepatocellular carcinoma^1^. Several risk factors linked to steatosis, including hepatitis C virus (HCV) infection, obesity, dysbetalipoproteinemia, amongst others^2^,^3^, are considered to have the potential to interfere with the balance between inflow (lipogenesis) and outflow (β-oxidation and lipid export) of lipid metabolism, which is under complicated and sophisticated controls, to maintain lipid homeostasis.

Blocking of lipoprotein assembly may inhibit lipid export, which is one of the causes of hepatic steatosis. The crucial role of microsomal triglyceride transfer protein (MTP) in the assembly and secretion of lipoproteins was originally identified in patients with abetalipoproteinemia, a syndrome caused by mutations in *MTP* gene^4^ and observed in MTP liver-specific knockout mice^5^. Recent study revealed that besides promoting lipogenesis, HCV infection also represses the expression of MTP through HCV viral proteins including core and nonstructural protein 5A (NS5A)^6^. Moreover, a polymorphism in the *MTP* promoter resulting in different MTP expression levels has been shown to be highly associated with chronic HCV infection which is a leading cause of hepatic steatosis^7^,^8^.

MTP, a key regulator of liver lipid homeostasis, is synthesized largely in the liver and intestine and is primarily modulated at the transcriptional level^9^. Extensive studies have revealed several transcription factors that modulate the MTP expression either positively or negatively and the existence of synergistic activation or inhibitory cross-talk on the *MTP* promoter mediated through physical interaction with these factors^9^. Additionally, a number of coactivators and corepressors also play key roles in the regulation of *MTP* gene expression^9^. Hepatocyte nuclear factor 4 (HNF4) is a highly conserved, liver-enriched nuclear receptor protein which can activate gene expression in the absence of exogenous ligands^10^. Numerous studies and genome-wide searches have revealed that HNF4 acts as a master transcription regulator which participates in the regulation or interaction with a broad range of potential target genes particularly for liver development, hepatocyte differentiation, lipid homeostasis and glucose metabolism^11^,^12^. The analysis of the liver-specific HNF4 null mice indicated that HNF4 is indispensable for the constitutive expression of MTP^13^. It is noted that HNF4-mediated transactivation on *MTP* promoter is inhibited by interaction with the corepressor, small heterodimer partner (SHP), which disrupts the formation of HNF4 homodimer and impedes its DNA binding ability^14^,^15^.

RNA helicase DDX3, also known as DBX or CAP-Rf^16^,^17^,^18^, contains a highly conserved core domain harboring RNA-dependent ATPase and ATP-dependent RNA helicase activities. Mounting evidence reveals that DDX3 is involved in a broad spectrum of RNA metabolism such as transcription, RNA splicing, mRNA export, translation and cytoplasmic mRNA granules^19^,^20^,^21^,^22^. Moreover, DDX3 has been reported to affect cell cycle progress, cell growth and tumorigenesis in a positive or negative manner^23^,^24^,^25^,^26^,^27^,^28^,^29^,^30^. A recent study indicated that DDX3 knockout mice were embryonic lethal^31^. Apart from acting as a multifunctional host cellular factor, DDX3 has been found to be involved in the HCV life cycle^32^,^33^,^34^. As noted, DDX3 was initially identified as an HCV core-interacting protein^16^,^17^,^18^, moreover, HCV core protein induces steatosis in HCV core-expressing transgenic mice^35^,^36^. Since HCV usually “hijacks” the host lipid metabolism to facilitate its multiplication and pathogenesis, DDX3 may play a role in lipid metabolism.

In this study, we found that downregulation of DDX3 inhibits the expression of MTP which in turn leads to lipid accumulation and affects ApoB secretion. DDX3 interacts with SHP and HNF4 *in vitro* and *ex vivo*. Moreover, DDX3 upregulates the *MTP* promoter by enhancing the binding of HNF4 to its response element, and disrupting the interaction between SHP and HNF4 to counteract the inhibitory effect of SHP. Together, these findings reveal a novel function of DDX3 in lipid homeostasis and a potential role of DDX3 on HCV-associated pathogenesis.

## Results

### HCV infection inhibits DDX3 and MTP expression leading to accumulation of intracellular triglyceride and reduction of secreted ApoB

**Figure 1:**
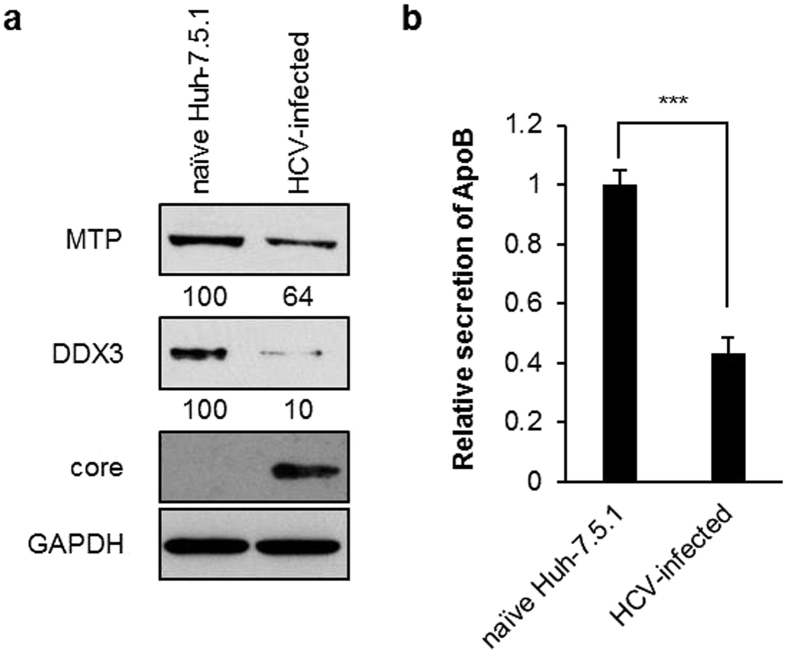
HCV infection suppresses DDX3, MTP expression and ApoB secretion. (**a**) HCV infection reduces the expression of DDX3 and MTP. The total cell extracts of HCV-infected/uninfected Huh-7.5.1 (see Materials and Methods) were collected and subjected to immunoblotting using anti-MTP (Santa Cruz Biotechnology), anti-DDX3^21^ and anti-core (Abcam) antibodies, and GAPDH was used as a loading control. Intracellular protein levels were quantified by ImageJ, normalized to GAPDH and represented as amount relative to that of uninfected Huh-7.5.1 cells. (**b**) HCV infection leads to reduced secretion of ApoB. Culture media harvested from HCV-infected/uninfected Huh-7.5.1 cells were subjected to ApoB detection by ELISA (see Materials and Methods). Results are represented as means ± S.D. for at least three independent experiments and ***indicates *p* < 0.001, is considered to be significant.

**Figure 2:**
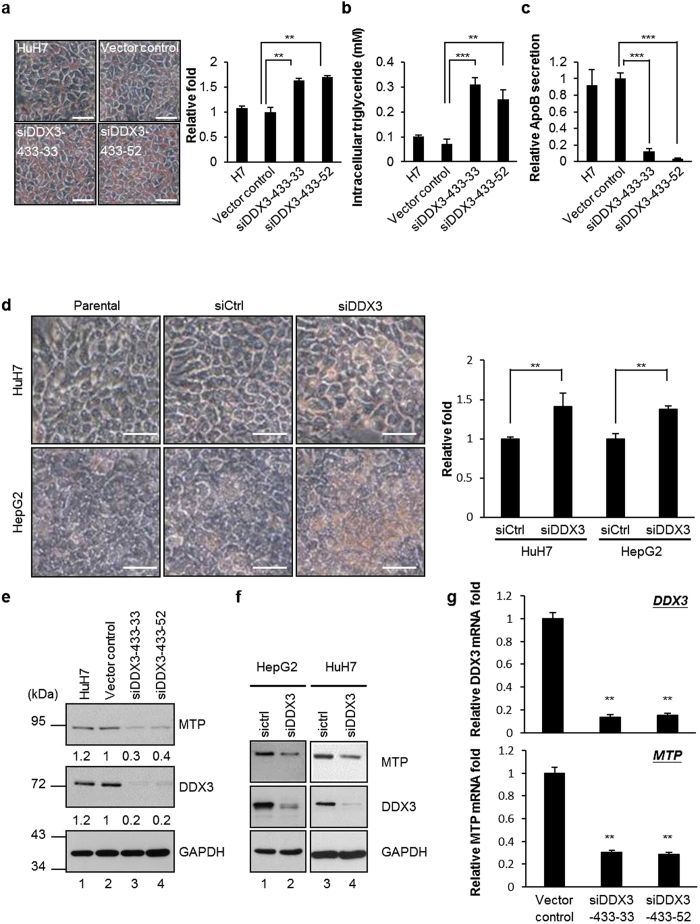
Knockdown of DDX3 inhibits the expression of MTP and leads to lipid accumulation. (**a**) Lipid accumulation was visualized by Oil Red O staining. The parental HuH7 cells, vector control and two DDX3 knockdown cell lines (siDDX3-433-33 and siDDX3-433-52) were fixed and stained with Oil Red O. The images were observed by light microscopy. Scale bars, 25 μm. Absorbance of isopropanol-eluted Oil Red O was then detected by a spectrophotometer. Results shown in the right panel was represented as the relative fold of the absorbance compared with the vector control. (**b** and **c**) DDX3 knockdown leads to accumulation of triglycerides and reduced secretion of ApoB. The cell lysates and culture medium of parental HuH7 cells, vector control and two DDX3 knockdown cell lines were subjected to detection of triglycerides and ApoB as described in Materials and Methods, respectively. Relative ApoB secretion in panel c is compared to the ApoB level of vector control cells. (**d**) HuH7 and HepG2 cells were transfected with control siRNA (siCtrl) or DDX3 siRNA (siDDX3). After 72 hr, cells were fixed, stained and Oil Red O absorbance was measured as described in panel a. (**e** and **f** ) DDX3 silencing represses the expression of MTP. The total cell extracts of two DDX3 knockdown cell lines, vector control, parental HuH7 as well as siCtrl/siDDX3 HuH7 and HepG2 cells were collected then subjected to immunoblotting using anti-MTP and anti-DDX3 antibodies, and GAPDH was used as a loading control. The relative fold of MTP and DDX3 amount in panel e was normalized by GAPDH and compared to those in vector control cells. (**g**) Detection of DDX3 and MTP mRNA levels by quantitative RT-PCR assay. Total cellular RNA was isolated from two DDX3 knockdown cell lines and vector control, then real time RT-PCR was performed with primers specific to mRNA of DDX3 or MTP. The GAPDH mRNA served as an internal control. Results shown in panel a,b,c,d and g are represented as means ± S.D. for at least three independent experiments. **p < 0.01; ***p < 0.001.

To explore the role of DDX3 in HCV pathogenesis, we first examined DDX3 protein expression in HCV infection. Interestingly, HCV infection severely inhibited the expression of DDX3 by 90% in parallel with reduced MTP protein levels to 64% as compared with uninfected cells (Fig. 1a). The secretion of ApoB by HCV-infected cells also declined by 57% as compared to uninfected Huh-7.5.1 cells, reflecting the decrease in MTP activity after HCV infection (Fig. 1b). MTP is a key regulator of lipid homeostasis. Therefore, the concurrent expression of the DDX3 and MTP proteins prompted us to examine whether DDX3 participates in the regulation of hepatic lipid metabolism via regulating MTP protein level. To this end, we performed Oil Red O staining experiments to detect the intracellular neutral triglycerides and lipids. Two stable DDX3 knockdown HuH7 cell lines, siDDX3-433-33 and siDDX3-433-52, together with the vector control and parental HuH7 cell lines were used. As shown in Fig. 2a (left panel), we found larger number red-colored particles located in these two DDX3 knockdown cell lines compared to the parental or vector control HuH7 cells. These findings were also validated by spectrophotometric quantification of 100% isopropanol eluted Oil Red O solution. The relative absorbance of eluted Oil Red O solution of two DDX3 knockdown cell lines were higher compared to the vector control or parental HuH7 cells (Fig. 2a, right panel, approximately 1.6 to 1.7-fold). The accumulation of triglycerides in DDX3 knockdown cell lines was further verified by directly assessing the intracellular triglyceride levels. As shown in Fig. 2b, both of the two DDX3 knockdown cell lines accumulated approximately 2.5 to 3-fold higher triglyceride than the vector control and parental HuH7 cells. Concomitantly, the secreted ApoB was also decreased by DDX3 knockdown as compared to the vector control and parental HuH7 cells (Fig. 2c, 87% to 97% reduction).

To further confirm these studies, transient transfection of DDX3 siRNA was conducted to knockdown endogenous DDX3 in HuH7 and HepG2 cells. A more clear and distinct form of the Oil Red O staining (Fig. 2d, left panel) and about 1.4-fold enhancement in absorbance of eluted Oil Red O from transient DDX3-knockdown cells (Fig. 2d, right panel) compared with parental and control siRNA-transfected cells were observed. These observations indicate that DDX3 knockdown increases the neutral lipid accumulation and suggest a potential role of DDX3 in hepatic lipid metabolism.

To investigate whether DDX3 modulates lipid metabolism through regulating MTP expression, we first determined the protein expression profiles of MTP in DDX3 knockdown cells. As shown in Fig. 2e, protein expression levels of MTP were dramatically reduced in DDX3 knockdown cell lines by 60–70% compared to control. Similar results were found in the transient siRNA-mediated DDX3 knockdown HuH7 and HepG2 cells (Fig. 2f). Consistently, mRNA expression levels of MTP were significantly diminished in DDX3 knockdown cell lines by 70% (Fig. 2g). The concomitant decrease of MTP protein expression and mRNA levels by DDX3 knockdown suggest that DDX3 may positively regulate MTP expression at the transcriptional level. Together, all these results indicate that DDX3 plays a role in regulation of MTP expression and imply that DDX3 may target MTP to regulate lipid homeostasis.

### DDX3 associates with SHP and HNF4 *in vitro* and *ex vivo*

**Figure 3:**
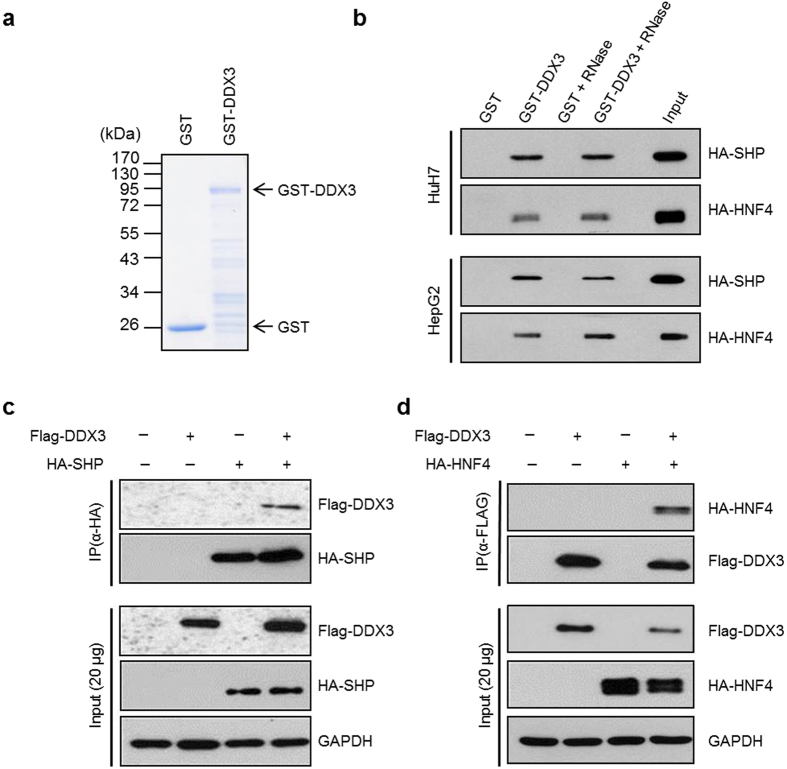
DDX3 interacts with SHP and HNF4 *in vitro* and *ex vivo*. (**a**) Coomassie brilliant blue staining of *E. coli* BL21 (DE3) expressed recombinant proteins, GST and GST-DDX3. (**b**) *In vitro* GST pull down assays. Purified GST and GST-DDX3 proteins prebound with glutathione-Sepharose 4B resins were incubated with nuclear extracts (200 μg) of HuH7 and HepG2 cells overexpressing HA-SHP or HA-HNF4 with or without RNase A treatment. Associated proteins were eluted and resolved by SDS-PAGE, then immunoblotted using anti-HA antibody (Roche). Input: nuclear extracts of HuH7 expressed HA-SHP (5%, 10 μg) or HuH7 expressed HA-HNF4 (2.5%, 5 μg). (**c** and **d**) *In vivo* co-immunoprecipitation assays. HuH7 cells were cotransfected with plasmids expressing Flag-DDX3 (10 μg) and HA-SHP (10 μg) (panel c) or with Flag-DDX3 (10 μg) and HA-HNF4 (10 μg) (panel d) expression constructs by calcium phosphate co-precipitation method. Nuclear extracts (200 μg) were isolated at 48 hr post-transfection and immunoprecipitated using anti-HA antibody- conjugated agarose beads (panel c) or anti-FLAG M2 affinity resins (panel d). The immunoprecipitates were analyzed by SDS-PAGE, then immunoblotting with anti-FLAG (Sigma) and anti-HA antibodies.

MTP gene expression is regulated primarily at the transcriptional level. Among the *MTP* promoter-targeting transcription factors, HNF4 is a critical positive regulator of MTP expression, while SHP inhibits the transactivation potential of HNF4 to down-regulate the expression of MTP^8^. In addition, our preliminary study by yeast two-hybrid screening revealed that SHP is one of the cellular proteins targeted by DDX3 (C. H. Chao and Y. H. Wu Lee, unpublished data). Therefore, to examine whether DDX3 modulates MTP expression through interaction with HNF4 and SHP, *in vitro* GST pull-down assays were performed. As shown in Fig. 3a and b, both HA-tagged SHP and HA-tagged HNF4 from nuclear extracts of HuH7 and HepG2 expressing HA-SHP and HA-HNF4 were pulled down by GST-DDX3 but not by GST control. The presence or absence of RNase A treatment has no influence on these interactions in both cell lines. To further verify the interaction between SHP, HNF4 and DDX3, *ex vivo* co-immunoprecipitation analyses were performed on RNase A-treated nuclear extracts of HuH7 cells co-expressing Flag-DDX3 alone or together with HA-SHP or HA-HNF4. In agreement with the *in vitro* pull down assay, Flag-tagged DDX3 and HA-tagged HNF4 were coimmunoprecipitated with HA-tagged SHP and Flag-tagged DDX3, respectively (Fig. 3c and d). Hence, these results showed that DDX3 associates with either SHP or HNF4 both *in vitro* and *ex vivo*, and this interaction suggests its functional importance in the regulation of MTP expression.

### DDX3 potentiates transactivation activity of HNF4 on MTP promoter

**Figure 4:**
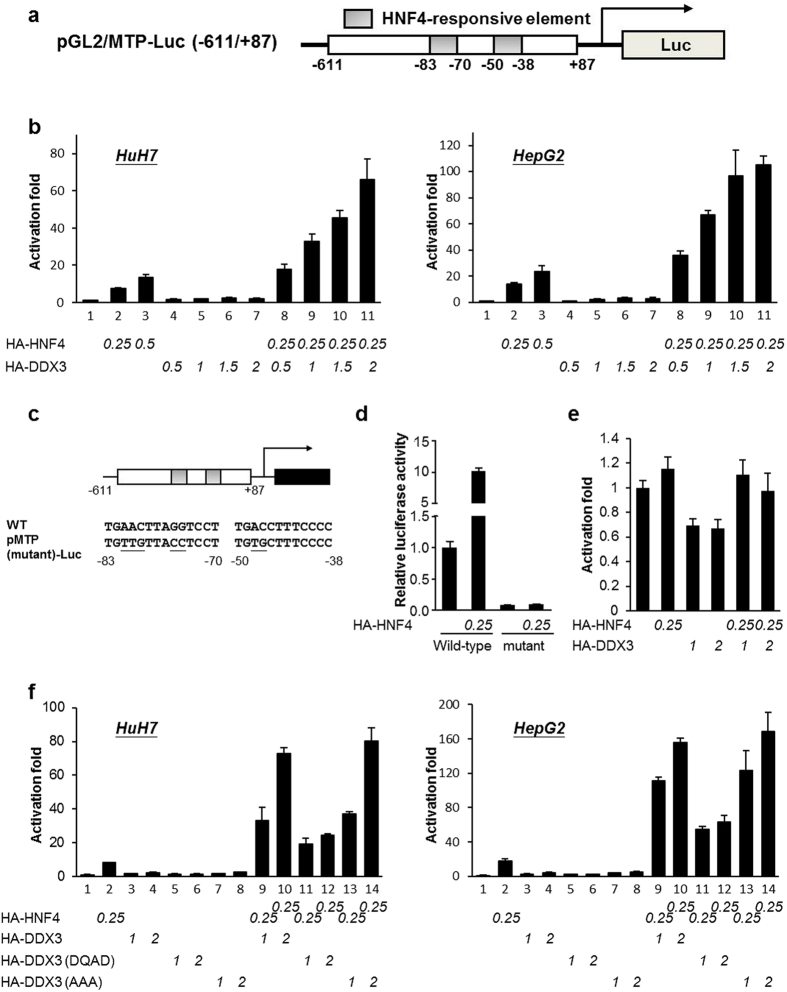
DDX3 up-regulates the HNF4-mediated transactivation of *MTP* promoter. (**a**) Schematic representation of pGL2/MTP-Luc (−611/+87) reporter plasmid. (**b**) DDX3 potentiates transactivation activity of HNF4 on *MTP* promoter. *MTP* promoter (−611~+87) -driven reporter plasmid (0.25 μg) was transfected alone or together with HA-HNF4 and HA-DDX3 expressing constructs in HuH7 and HepG2 by lipofectamine 2000. The total amount of transfected plasmids was adjusted to 2.5 μg/35 mm dish by supplementing with control vector, pcDNA/HA. After 48 hr, cells were collected and subjected to luciferase activity assay. (**c**) Schematic representation of pMTP(mutant)-Luc reporter plasmid, with the mutated sequences specified (underlined). (**d**) Destruction of HNF4-responsive elements blocks transactivation activity of HNF4 on MTP promoter. pGL2/MTP-Luc (−611/+87) reporter plasmid (Wild-type; 0.25 μg) or pMTP(mutant)-Luc plasmid (mutant; 0.25 μg) was transfected alone or together with HA-HNF4 expressing construct (0.25 μg) as indicated in HuH7 by lipofectamine 2000. The total amount of transfected plasmids was adjusted to 2.5 μg/35 mm dish by supplementing with control vector, pcDNA/HA. Forty-eight hours later, cells were collected and subjected to luciferase activity assay. (**e**) Destruction of HNF4-responsive elements blocks synergistic transactivation activity of HNF4/DDX3 on MTP promoter. pMTP(mutant)-Luc reporter plasmid (0.25 μg) was transfected alone or together with HA-HNF4 and HA-DDX3 expressing constructs as indicated amount in HuH7 and HepG2 by lipofectamine 2000. The total amount of transfected plasmids was adjusted to 2.5 μg/35 mm dish by supplementing with control vector, pcDNA/HA. Cells were collected and subjected to luciferase activity assay 48 hr post-transfection. The relative luciferase activity of pGL2/MTP-Luc (−611/+87) reporter alone is arbitrarily taken as one. All data are represented as the average (mean ± S.D.) of at least three independent experiments. (**f**) ATPase activity of DDX3 is required for its transactivation activity of HNF4 on *MTP* promoter. HuH7 and HepG2 cells were transfected with pGL2/MTP-Luc (−611/+87) reporter plasmid (0.25 μg) alone or together with indicated amount of plasmids expressing HA-HNF4, HA-DDX3(WT), HA-DDX3(DQAD) and HA-DDX3(AAA). Luciferase activity was measured 48 hr post-transfection.

To delineate the DDX3-mediated transactivation of HNF4 on *MTP* promoter, luciferase reporter assays were carried out. A fixed amount of reporter plasmid pGL2/MTP-Luc(−611/+87) (illustrated in Fig. 4a) alone or together with various amounts of expression plasmids for HA-DDX3 and HA-HNF4 were cotransfected into HuH7 cells. Consistent with the previous report^37^, transient expression of HNF4 alone activated *MTP* promoter activity and was in a dose-dependent manner (Fig. 4b *HuH7*, lanes 2 and 3). By contrast, overexpression of DDX3 alone only had moderate effects on pGL2/MTP-Luc reporter activity (Fig. 4b *HuH7*, lanes 4–7). However, cotransfection of HA-HNF4 and HA-DDX3 expression constructs into HuH7 cells significantly enhances the transcriptional activity of HNF4 on *MTP* promoter by 2.4- to 8.9-fold in a DDX3 dose-dependent manner (Fig. 4b *HuH7*, lane 8–11 versus lane 2). These results indicate that DDX3 interacts with HNF4 to synergistically transactivate the *MTP* promoter activity. Similar results were also observed in HepG2, HCT116 and HeLa cells (Fig. 4b and Supplementary Fig. S1, lanes 8–11 versus lane 2), suggesting that DDX3 utilizes a similar mechanism to potentiate the transcriptional activity of HNF4 on *MTP* promoter independent of cell type. DDX3 has been shown to promote translation of mRNA containing highly structured 5′ UTR^38^,^39^. Given that the pGL2/MTP-Luc(−611/+87) reporter plasmid harbors a part of the highly structured MTP 5′ UTR, we then examined whether DDX3/HNF4 exerts their transactivating function by promoting translation of the reporter mRNA. To this end, reporter assays were performed with the pMTP(mutant)-Luc reporter plasmid in which both HNF4-responsive elements were mutated (Fig. 4c). As shown in Fig. 4d, destruction of both HNF4-responsive elements largely reduced the luciferase activity of pMTP(mutant)-Luc and the transactivation activity of HNF4 on pMTP(mutant)-Luc. Moreover, no synergistic effects of HNF4/DDX3 were observed with HNF4-responsive element-mutated reporter (Fig. 4e), confirming that HNF4-responsive elements are essential for synergistic transactivation of the MTP promoter by HNF4 and DDX3.

DDX3 has been shown to possess ATPase and RNA helicase activities. To elucidate whether DDX3 required its ATPase and RNA helicase activities to transactivate the *MTP*, luciferase reporter assays were performed. The expression constructs of DDX3/DQAD mutant (abolishes both ATPase and ATPase-dependent helicase activities) and DDX3/AAA mutant (impairs RNA unwinding activity only but retains the ATPase activity of the helicase)^40^ were introduced individually into HuH7 and HepG2 cells. As shown in Fig. 4f, in the presence of exogenously expressed HNF4, the DDX3/AAA mutant up-regulated HNF4-response reporter activity to a similar level as wild type DDX3 in both HuH7 cells (Fig. 4f, DDX3/WT: lanes 9 and 10 versus lane 2; DDX3/AAA: lanes 13 and 14 versus lane 2) and HepG2 cells (Fig. 4f, DDX3/WT: lanes 9 and 10 versus lane 2; DDX3/AAA: lanes 13 and 14 versus lane 2) in a dose-dependent manner. Conversely, compared to the DDX3 wild type, DDX3/DQAD mutant reduced the reporter activity to 34–59% in HuH7 cells and 41–49% in HepG2 cells (Fig. 4f, lane 12 versus lane 10 and lane 11 versus lane 9). Although there were no significant effects on *MTP* promoter in the absence of transfected HNF4, DDX3/AAA mutant alone showed a comparable level of the reporter activity to wild type DDX3. In addition, DDX3/DQAD mutant alone diminished the reporter activities to 59–81% and 46–74% (Fig. 4f, HuH7 and HepG2, respectively) of that in wild type DDX3. The protein expression levels were similar among DDX3 wild type and two mutant constructs (Fig. S2a) and the exogenous HNF4 protein levels were not altered by cotransfection of DDX3-expressing plasmid (Fig. S2b). These results provide strong evidences that DDX3 promotes transactivation activity of HNF4 on *MTP* promoter through its ATPase activity.

### DDX3 enhances the recruitment of HNF4 to the MTP promoter

**Figure 5:**
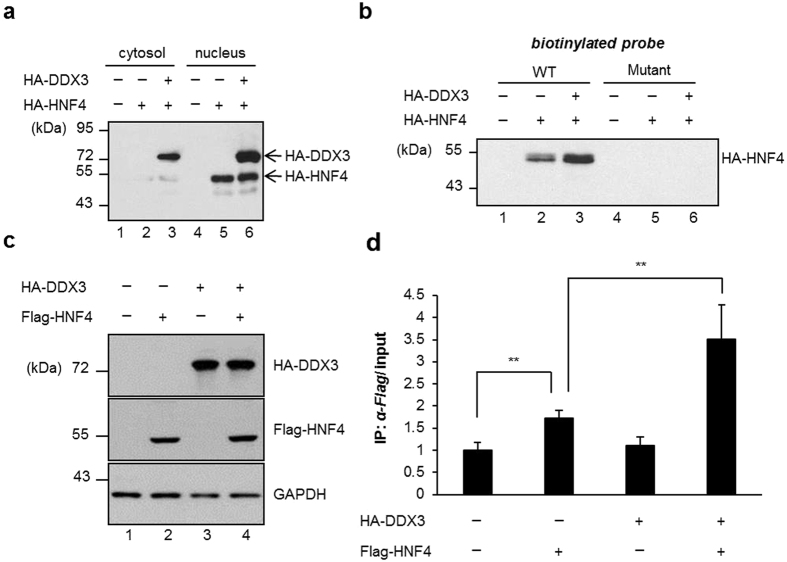
DDX3 enhances HNF4 binding to the *MTP* promoter. (**a**) Western blot analysis of subcellular localization of DDX3. HuH7 cells were transfected with plasmid expressing HA-HNF4 alone or together with HA-DDX3 expression construct as indicated. After 48 hr, cytosolic fractions and nuclear extracts (20 μg of each) were prepared and subjected to SDS-PAGE then immunoblotting with anti-HA antibody. (**b**) HNF4 DNA binding ability is enhanced by DDX3. The nuclear extracts of transfected HuH7 cells were incubated with annealed biotinylated probe containing HNF4-responsive element and subjected to binding with streptavidin agarose. After extensive washing, the bound fractions were analyzed by SDS-PAGE, followed by immunoblotting with anti-HA antibody. (**c**) Western blot analysis of the ectopically expressed Flag-HNF4 and HA-DDX3. HuH7 cells were transfected with plasmids expressing HA-DDX3 and Flag-HNF4 as indicated. Western blot analysis was performed 48 hr posttransfection. The expression level of HA-DDX3 and Flag-HNF4 was detected using anti-HA and anti-Flag antibodies, respectively. (**d**) Overexpression of DDX3 enhances the DNA binding activity of Flag-HNF4 on *MTP* promoter. Chromatin immunoprecipitation assay was performed by isolation of soluble chromatin fragments prepared from transfected-HuH7 cells and then immunoprecipitated with anti-FLAG M2 agarose resins. Immunoprecipitates were analyzed by quantitative real-time PCR with specific primers for *MTP* promoter (as described in Materials and Methods). Results are expressed as means ± S.D. for at least three independent experiments and **p < 0.01, is considered to be significant.

To elucidate whether DDX3 enhances the transactivation activity of HNF4 through increasing the DNA-binding affinity of HNF4 on its responsive element, streptavidin-biotin agarose affinity chromatography was performed. As shown in Fig. 5a and b, a HNF4-specific binding signal in nuclear extracts prepared from HuH7 cell expressing HA-HNF4 alone was about 3-fold lower in comparison with HA-HNF4 together with HA-DDX3 (Fig. 5b, lane 3 versus lane 2). In contrast, the experiments performed in mutant biotinylated probe (Fig. 5b, lanes 4–6) and vector control with wild type biotinylated probe (Fig. 5b, lane 1) had no detectable signals, indicating that the binding signals were sequence-specific and DDX3 enhanced HNF4 binding to its responsive element *in vitro*.

To further confirm the increased of HNF4 DNA-binding ability by DDX3 *ex vivo*, ChIP-qPCR analysis was performed to elucidate HNF4 binding affinity on *MTP* promoter. Cotransfection of HA-DDX3 and Flag-HNF4 expressing constructs increased the recruitment of Flag-HNF4 to the *MTP* promoter in comparison with exogenously expressed Flag-HNF4 alone (Fig. 5d, 3.5-fold versus 1.7-fold, lane 4 and lane 2 versus lane 1, respectively). Exogenously expressed DDX3 had no impact on the protein level of HNF4 (Fig. 5a, lane 5 versus lane 6, and Fig. 5c, lane 2 versus lane 4). This is in agreement with our previous result (Fig. S2b). Taken together, our results demonstrate that DDX3 enhances the recruitment of HNF4 to the *MTP* promoter *in vitro* and *ex vivo*.

### DDX3 interacts with CBP/p300 and induces acetylation of HNF4

**Figure 6:**
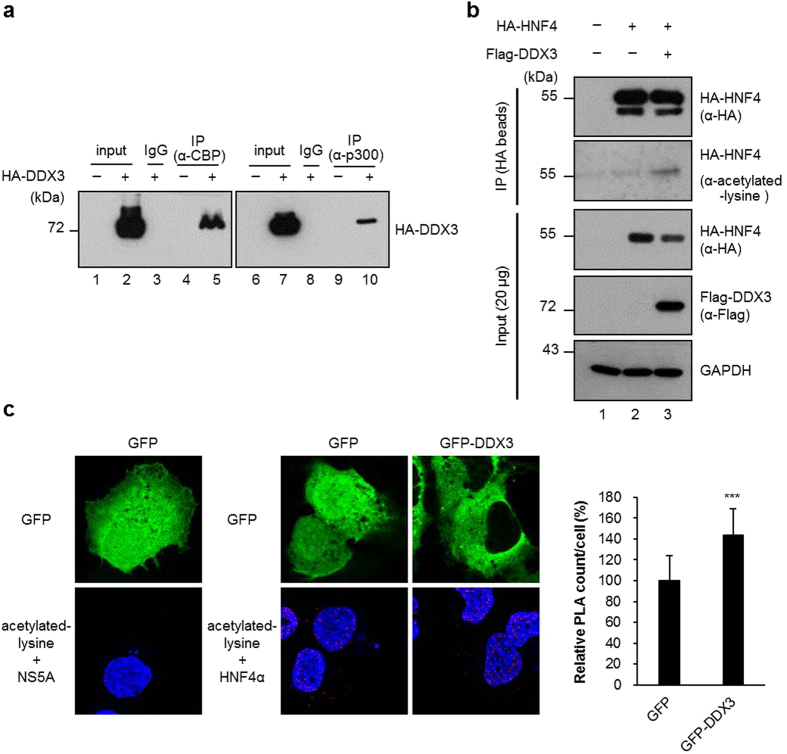
DDX3 interacts with CBP/p300 and induces the acetylation status of HNF4. (**a**) DDX3 interacts with CBP and p300 *in vivo*. HuH7 cells were transfected with HA-DDX3 expressing plasmid or vector control. After 48 hr, the nuclear fractions (200 μg) were collected and incubated with antibodies against CBP or p300, and then the mixtures were bound to protein G sepharose beads. The precipitates were subjected to immunoblotting with anti-HA antibody. The IgG-conjugated protein G sepharose beads (mouse, lane 3 and lane 8) incubated with HA-DDX3 expressed nuclear extracts were used as negative control. Input; 10% (20 μg) of the nuclear extract. (**b**) Ectopic expression of DDX3 induces the acetylation status of HA-HNF4. HuH7 cells were transfected with plasmid expressing HA-HNF4 alone or together with HA-DDX3 expression construct as indicated. The whole cell extracts (1 mg) prepared from the transfected cells were subjected to immunoprecipitation of HA-HNF4 with anti-HA agarose beads. After extensive wash, the total amounts and acetylated forms of immunoprecipitated HA-HNF4 were detected by Western blot analysis with anti-HA and anti-acetylated lysine (Cell Signaling Technology) antibodies, respectively. (**c**) *In situ* proximity ligation assay (PLA) indicates that ectopic expression of DDX3 induces endogenous HNF4 acetylation. HuH7 cells transfected with either GFP or GFP-DDX3 expressing plasmids for 48 hr were fixed and subjected to PLA with anti-HNF4 (Abcam) and anti-acetylated lysine antibodies. Anti-NS5A antibody (Austral Biologicals) served as control. Nuclei were stained with DAPI (blue). The numbers of PLA signals per cell were quantified using MetaMorph software (Molecular Devices) and are shown as means ± S.D. relative to that of GFP-transfected cells. n = 50. ***p < 0.001.

CREB-binding protein (CBP) is known to interact with and induce acetylation of HNF4^41^, which is critical for the nuclear retention and the DNA-binding activity of HNF4^42^. This prompted us to investigate whether DDX3 interacts with CBP/p300 to induce HNF4 acetylation thereby enhancing the binding of HNF4 to the *MTP* promoter. To test this possibility, we first performed the co-immunoprecipitation assay with nuclear extracts from HuH7 transiently expressing HA-DDX3. As shown in Fig. 6a, HA-DDX3 was coimmunoprecipitated by both anti-CBP antibody-conjugated (lane 5) and anti-p300 antibody-conjugated (lane 10) beads, but not by IgG controls (lanes 3 and 8). This result implies that DDX3 associates with CBP and p300 to form a complex *ex vivo*.

Next, we examined whether the acetylation status of HNF4 is enhanced in the presence of exogenously expressed DDX3. As shown in Fig. 6b, the amounts of immunoprecipitated HA-HNF4 were comparable between HuH7 cells expressing HA-HNF4 alone and together with Flag-DDX3 (top, lanes 2 and 3). However, acetylation of HA-HNF4 were apparently enhanced in HuH7 cells co-expressing HA-HNF4 and Flag-DDX3 (Fig. 6b, detected by anti-acetylated-lysine antibody, compare lane 3 to lane 2). The acetylation-promoting role of DDX3 in HNF4 acetylation was further confirmed by the *in situ* proximity ligation assay (PLA) of GFP and GFP-DDX3 expressing HuH7 cells. Overexpression of GFP-DDX3 significantly enhanced acetylation of HNF4 by 44% as compared with GFP expressing cells (Fig. 6c). The above findings support the notion that DDX3 interacts with CBP/p300 and enhances the acetylation status of HNF4 thereby increasing the DNA-binding affinity of HNF4 to its response element.

### DDX3 partially relieves the SHP-mediated suppression on MTP promoter

**Figure 7:**
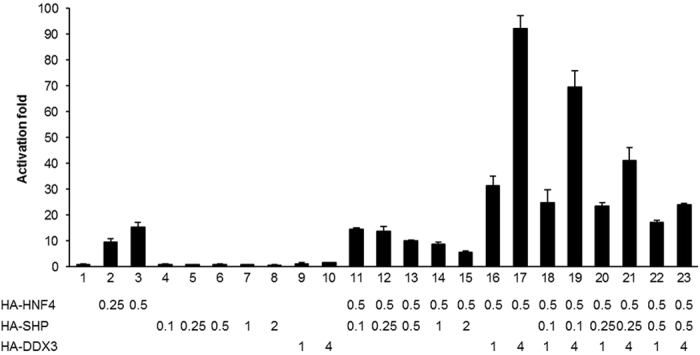
DDX3 partially relieves the SHP-mediated repression effect on *MTP* promoter. HuH7 cells were transfected with pGL2/MTP-Luc(−611/+87) reporter plasmid (0.5 μg) alone or together with a combination of indicated amount of HA-HNF4, HA-SHP and HA-DDX3 expression plasmids. After 48 hr, cells were harvested and lucifearse activity assay was performed. The total amount of transfected plasmids was kept constant by adding control vector, pcDNA/HA. The relative luciferase activity is represented as activation fold compared to basal expression level of the MTP reporter plasmid alone (lane 1). Results are represented as the average (mean ± S.D.) of at least three independent experiments.

Since DDX3 interacts with both HNF4 and SHP (Fig. 3), and they both play vital roles in positive or negative modulation of MTP promoter activity, we next examined the regulatory cross-talk among DDX3, HNF4 and SHP. Therefore, transient luciferase reporter assays were performed by cotransfecting a fixed amount of reporter plasmid pGL2/MTP-Luc(−611/+87) alone or together with indicated amounts of expression constructs encoding HA-DDX3, HA-HNF4 and HA-SHP into HuH7 cells. Transient expression of HNF4 alone lead to a dose-dependent activation of *MTP* promoter activity from 9.6- to 15.2-fold (Fig. 7, lanes 2 and 3 versus lane 1). In the presence of a fixed quantity of exogenous HNF4, the reporter activities were either suppressed to 37% or upregulated to 6-fold by increasing amounts of ectopically expressed HA-SHP (Fig. 7, lanes 11–15 versus lane 3) or HA-DDX3 (Fig. 7, lanes 16–17 versus lane 3), respectively. When all three proteins are coexpressed, both low (Fig. 7, lanes 18, 20 and 22 compared to lanes 11, 12 and 13) and high (Fig. 7, lanes 19, 21 and 23 compared to lanes 11, 12 and 13) expression levels of exogenous DDX3 could partially relieve the SHP-mediated suppressive effect on *MTP* promoter to different extent. These results strongly suggest that the DDX3 indeed interacts with HNF4 and SHP to regulate the *MTP* promoter.

### DDX3 disrupts the inactive SHP/HNF4 heterodimer formation and promotes the formation of the active HNF4 homodimer

**Figure 8:**
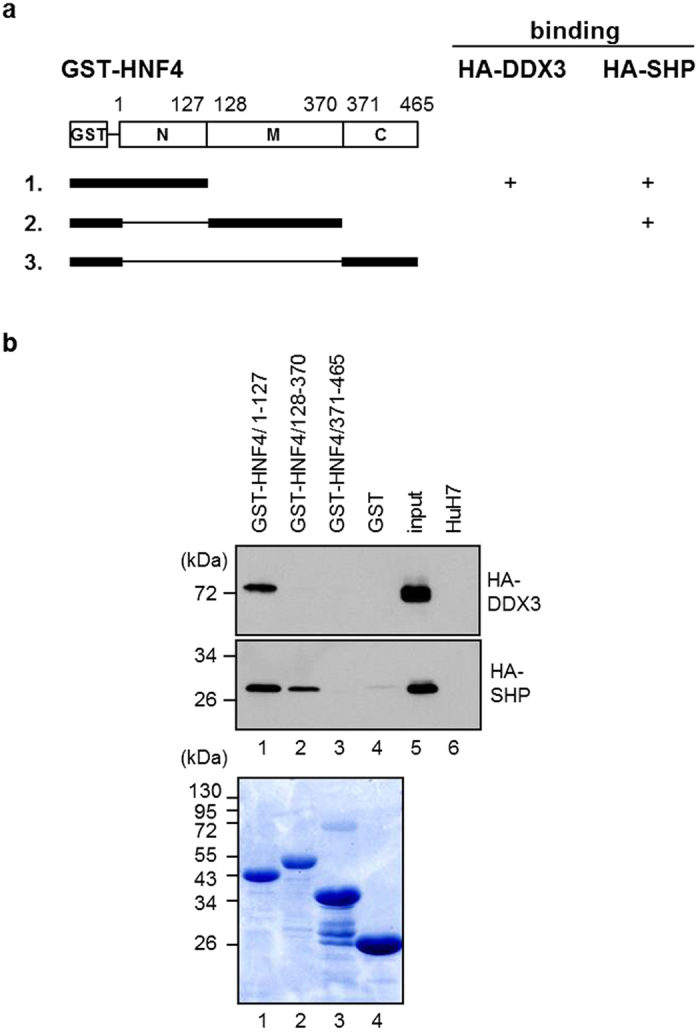
HNF4 interacts with DDX3 through one of its SHP-interacting domains. (**a**) Schematic representation of the GST fusion proteins containing N-terminal, middle and C-terminal regions of HNF4. The binding results of the GST pull down analysis shown in Fig. 6b are summarized. (**b**) Mapping the interaction domains of DDX3 and SHP with HNF4. The GST and GST-HNF4 truncated derivatives prebound with glutathione Sepharose beads were incubated with whole cell lysates (500 μg) from HuH7 cells expressing HA-DDX3 and HA-SHP, respectively. After extensive wash, the bound proteins were resolved by SDS-PAGE and analyzed by Western blot with anti-HA antibody. Lane 5, input: total cell lysate (20 μg) of HuH7 cells expressing HA-tagged DDX3 or HA-tagged SHP. Lane 6, total cell lysate (20 μg) of HuH7 cells. Coomassie brilliant blue staining of the GST-HNF4 truncated derivatives is shown in the lower panel.

**Figure 9:**
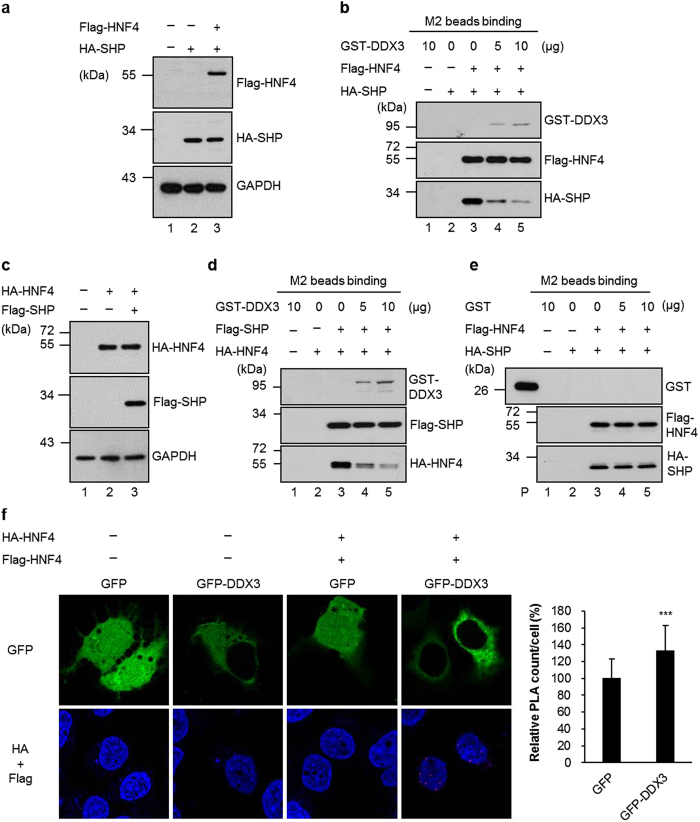
DDX3 disrupts the formation of SHP/HNF4 heterodimer and promotes the formation of the active HNF4 homodimer. (**a** and **b**) HuH7 cells were transfected with HA-SHP expression plasmid alone or together with Flag-HNF4 expression plasmid as indicated. (**a**) Immunoblotting was performed with antibodies against HA and Flag. (**b**) The total cell lysates (2 mg) of co-expressed Flag-HNF4 and HA-SHP were incubated with anti-Flag M2 agarose resins. After extensive washing, increasing amounts of purified GST-DDX3 proteins (as indicated on the top, lane 3–5) were added to the mixtures of the beads with bound fractions. The immunoprecipitated proteins were analyzed by immunoblotting with antibodies against Flag, HA and DDX3. The GST-DDX3 alone (lane 1) and cell lysates of expressed HA-tagged SHP (lane 2) incubated with anti-Flag M2 agarose resins were used as negative control. (**c**,**d** and **e**) Similar experiments were performed as shown in panel a and b, except HuH7 cells were transfected with HA-HNF4 expression plasmid alone or together with Flag-SHP expression plasmid (**c** and **d**), or purified GST protein was used instead of GST-DDX3 in (**e**). (**f**) Overexpression of DDX3 increases HNF4 homodimer formation. HuH7 cells were transfected with either GFP or GFP-DDX3 expressing plasmids together with HA and Flag vectors (−) or expressing plasmids for HA-HNF4 and Flag-HNF4 (+). Forty-eight hours later, cells were fixed and analyzed by PLA with anti-HA (Abcam) and anti-Flag antibodies. Nuclei were stained with DAPI (blue). In cells cotransfected with HA-HNF4 and Flag-HNF4, the numbers of PLA signals per cell were quantified using MetaMorph software and are shown as means ± S.D. relative to that of GFP-cotransfected cells. n = 50. ***p < 0.001.

The active form of HNF4 is a homodimer, and SHP has been shown to repress HNF4 transactivation via interaction with N-terminal and middle region of HNF4 (Fig. 8a) to prevent HNF4 homodimer formation and block its DNA-binding ability^14^. To address the underlying mechanisms by which DDX3 affects HNF4 and SHP on *MTP* promoter, we further delineated the interaction among DDX3, SHP and HNF4 by GST-pull down assay. As shown in Fig. 8b, DDX3 associated with HNF4 at N-terminal region, one of the two binding regions of HNF4 with SHP. Since DDX3 also associated with SHP (Fig. 3b and c), this raised the possibility that DDX3 might have the potential to disrupt the inactive SHP/HNF4 heterodimer formation. Therefore, competition assay was performed. Cell extracts from HuH7 cells co-expressing HA-SHP and Flag-HNF4 were preincubated with anti-Flag M2 beads, and addition of an increasing amount of purified GST-DDX3 (0, 5 and 10 μg) resulted in a dose-dependent blockage of HA-SHP binding to Flag-HNF4, while the GST-DDX3 was retained on it (Fig. 9b, lanes 3–5). Similarly, reciprocal competition assay also showed that GST-DDX3 impeded HA-HNF4 binding to Flag-SHP (Fig. 9d, lanes 3–5). To exclude signals from non-specific binding of proteins to the resins, we also examined the purified GST-DDX3 alone (Fig. 9b and d, lane 1) and cell lysates of HA-tagged SHP (Fig. 9b, lane 2) or HA-tagged HNF4 (Fig. 9d, lane 2) incubated with anti-Flag M2 beads. No false positive signals were observed. In addition, the purified GST protein has no impact on HA-SHP associated with Flag-HNF4 (Fig. 9e).

To examine whether the formation of the active HNF4 homodimer *ex vivo* was affected by DDX3 competing with SHP to bind HNF4, the *in situ* proximity ligation assay (PLA) was performed using HuH7 cells co-expressing either GFP or GFP-DDX3 together with HA-HNF4 and Flag-HNF4. The HA-HNF4/Flag-HNF4 interaction was increased by overexpression of GFP-DDX3 by 32% as compared with that of GFP-expressing cells (Fig. 9f). Detection of the specific interaction between HA-HNF4 and Flag-HNF4 by PLA was confirmed with HuH7 cells transfected with either GFP or GFP-DDX3 alone (Fig. 9f). These results indicate that DDX3 disrupts the interaction between HNF4 and SHP to form inactive HNF4/SHP heterodimer and enhanced the formation of active HNF4 homodimer. Collectively, our results demonstrate that DDX3 promotes MTP expression by increasing HNF4 acetylation and enhancing homodimer formation leading to its recruitment to the *MTP* promoter.

## Discussion

There is increasing evidence that RNA helicase DDX3 participates in almost all aspects of RNA metabolism and acts as a multifaceted protein to engage in diverse cellular processes^21^,^22^,^28^. Recently, DDX3 has attracted more attention as a result of its relevance to viral replication, tumorigenesis and innate immune response. Here, we provide new insights into the regulatory function of DDX3 in lipid metabolism. Using Oil Red O staining, triglyceride measurement and secreted ApoB detection, we observed that down-regulation of DDX3 led to lipid accumulation and reduced ApoB secretion in hepatocyte-derived HuH7 orHepG2 cell lines (Fig. 2a and d). The mRNA and protein expression profiles of MTP, a key factor in maintenance of lipid homeostasis, were repressed by siRNA-mediated knockdown of DDX3 (Fig. 2e–g). We further demonstrated that DDX3 interacted with HNF4 and SHP (Fig. 3) *in vitro* and *ex vivo,* and up-regulated *MTP* promoter activity in a cell type-independent manner (Fig. 4b and Supplementary Fig. S1) through increasing HNF4 acetylation (Fig. 6b and c) and HNF4 homodimer formation (Fig. 9f) while disrupting the inhibitory HNF4/SHP heterodimer formation (Fig. 9b and d).

Acetylation of HNF4 enhances its DNA binding ability on its response element and increases HNF4-targeted gene expression while deacetylation of HNF4 reduces its binding affinity on target promoter^42^,^43^. Here, we found that overexpression of DDX3 elevated acetylation of HNF4 throughout the nucleus (Fig. 6b and c), supporting the notion that DDX3 may not only has the potential to up-regulate *MTP* promoter activity by enhancing HNF4 DNA binding ability (Fig. 5b and d) but may also activate other HNF4 target promoters globally. In this study, our results indicated that DDX3 competed with SHP for binding to HNF4 disrupting the inactive HNF4/SHP heterodimer formation and reducing the suppressive effect of SHP on HNF4 homodimer binding to *MTP* promoter (Fig. 9b and d). Since HNF4 has been reported to be a master hepatic transcription factor, the DDX3-mediated induction of HNF4 acetylation and HNF4 homodimer formation (Fig. 9f) may partially be responsible for the multifunctional roles of DDX3.

DDX3 interacts with the histone acetyltransferases, CBP/p300 (Fig. 6a), and may regulate chromatin remodeling around the *MTP* promoter. It is well established that DEAD box RNA helicase family proteins exhibit ATPase and RNA helicase activities. In this study, the effect of DDX3 on HNF4-mediated transactivation of *MTP* promoter was largely dependent on the ATPase but not the RNA helicase activity (Fig. 4f). However, because DDX3 associates with the global coactivators CBP and its functional homolog p300 (Fig. 6a), the DDX3(DQAD) mutant with disrupted ATPase and RNA helicase activities may still have the ability to recruit coactivators to partially transactivate *MTP* promoter activity (Fig. 4f, lanes 11 and 12 versus lane 2). Whereas the chromatin remodeling is an ATP consuming process^44^, DDX3 might participate in both histone modification and chromatin remodeling or even coordinate these two processes through its ATPase activity and its interaction with different histone modifiers and chromatin remodeling complexes.

DDX3 has been reported to associate with various transcription factors to either activate or repress different promoters, including p21waf1/cip1^23^ and E-cadherin^45^ promoters. Our recent study has shown that DDX3 can even regulate gene transcription epigenetically. In hepatoma cells, DDX3 maintains the transcription of a subset of tumor-suppressive miRNA, including miR-122, by inhibiting the recruitment of DNMT3A to the target promoters leading to reduced hypermethylation on these miRNA promoters^25^. Hence, it is possible that DDX3 may also activate MTP expression epigenetically. Additionally, given that miR-122 itself is a target gene of HNF4 46, DDX3 may also activate miR-122 transcription through cooperating with HNF4 on the miR-122 promoter. MiR-122 is the most plentiful microRNA in the liver^47^,^48^. Knockout of miR-122 in mice leads to downregulation of MTP and steatohepatitis^49^. Notably, the fact that DDX3 positively regulates miR-122, which maintains MTP expression, reveals that DDX3 may not only augment MTP levels through directly transactivating *MTP* promoter but may also elevate MTP expression via manipulating the upstream regulatory factors. Our studies together with the previous reports hence reveal a complex network controlling the expression of MTP as well as the delicate cellular regulation of lipid homeostasis.

In this study, our data showed that DDX3 up-regulates *MTP* promoter and knockdown of DDX3 results in lipid accumulation and reduced ApoB secretion. However, the clinical relevance and physiological significance of DDX3 on *MTP* promoter have yet to be fully elucidated. Clinical reports have indicated that occurrence of liver steatosis in HCV patients is obviously higher than patients with other forms of chronic liver disease^50^,^51^. A series of studies have demonstrated that HCV viral proteins, core and NS5A, interfered with host lipid metabolism and resulted in lipid accumulation^35^,^52^,^53^. In particular, inhibition of MTP is one of the most common targets among these factors. All these reports implied a direct correlation between HCV and liver steatosis. Given that DDX3 interacts with HCV core and NS5A^16^,^17^,^18^,^34^ and is down-regulated by HCV infection (Fig. 1a), HCV infection may interfere with the DDX3-augmented transactivation activity of HNF4 on *MTP* promoter leading to reduced MTP expression by which, at least in part, HCV infection induces liver steatosis. The proteomic analysis of isolated HCV lipo-viral particles indicated that the particles were enriched in apolipoprotein B, apolipoprotein E and MTP^54^,^55^,^56^. More evidences also support that MTP activity and lipoprotein assembly is required for HCV viral assembly and secretion^57^,^58^. Clinical observations identified a functional genetic polymorphism −493G/T in the *MTP* promoter, where the T allele associates with higher MTP transcription level^59^. Intriguingly, even with reduced MTP mRNA levels and MTP activity, most chronic HCV-infected patients carry the T allele^8^,^60^. The authors have proposed that the host cells with −493T polymorphism in *MTP* promoter is a more favorable target for HCV multiplication in the early stage of infection. While at the later stage of infection, suppression of MTP expression is beneficial for the “storage” of HCV virus particles in lipid droplets for its latency and persistent infection^61^. Interestingly, DDX3 was identified as a cellular cofactor of HCV infection^32^,^33^,^34^,^62^,^63^,^64^. From this point of view, it makes sense that HCV hijack DDX3 to regulate *MTP* promoter to aid its virus propagation and persistent infection.

Besides being a HCV cellular cofactor^32^,^33^,^34^,^62^,^63^,^64^, DDX3 has also been demonstrated to participate in antiviral innate immunity^65^ and translocate to the HCV-induced stress granules in HCV infected cells^63^. It has been shown that the polyU/UC tract of HCV 3′ NTR induces DDX3-mediated IPS activation which promotes the expression of INF-β^66^. Moreover, recent study indicated that DDX3 promotes the translation of the PACT mRNA to enhance antiviral innate immunity^67^. Although both of these pathways can be abrogated by HCV core protein, it in turns highlights the important roles of DDX3 in antiviral innate immunity. Since DDX3 plays multifaceted roles in HCV infection, an elevated level of DDX3 may improve HCV replication directly but may also induce innate immunity that inhibit HCV propagation. Moreover, DDX3 associates with HCV 3′ NTR and IKK-α in the stress granules in HCV infection^63^. Proteins that aggregate in stress granules are more insoluble^68^, it may be one of the reasons why we detected lower DDX3 levels in HCV infected cells. The sequestration of DDX3 in stress granules may abrogate its transactivation activity on the MTP promoter in the nucleus and its translational regulation in the cytoplasm^19^,^38^,^39^. Similarly, although MTP is also a proven HCV cellular cofactor^57^, both we (Fig. 1a) and others^6^ demonstrated that expression of MTP is down-regulated during HCV infection.

HCV infection and steatosis are risk factors of hepatocellular carcinoma (HCC). Our recent study has shown that down-regulated DDX3 levels are correlated with high tumor grade and poor prognosis in HCC patients, and its reduction causes down-regulation of tumor-suppressive miR-122^25^. Accordingly, a series of studies have revealed the tumor-suppressive role of DDX3 in HCC^23^,^24^,^25^. However, DDX3 has been shown to act as either tumor suppressor or oncogene and even both in a rare case in various tumor types^28^. Here, we report that DDX3 synergistically increases HNF4-mediated transactivation activity via promoting HNF4 acetylation and enhancing homodimer formation. Given that both HNF4 and miR-122 are crucial factors suppressing hepatic tumorigenesis, our studies offer a novel aspect for the tumor suppressor role of DDX3 in HCC.

Hepatic steatosis is often associated with the development of hepatic fibrosis or cirrhosis and may even lead to hepatocellular carcinoma. Recently, more approaches focused on MTP as a therapeutic candidate in virtue of its physiological importance of lipoprotein secretion. Our findings demonstrate a novel role of DDX3 in lipid metabolism and provide a clue to design new strategies for therapy of hepatosteatosis.

## Materials and Methods

### Cell culture and transfection

Human hepatocellular carcinoma HuH-7, Huh-7.5.1^69^ (kindly provided by Dr. Francis V. Chisari, Scripps Research Institute), human hepatocellular blastoma HepG2, human cervical carcinoma HeLa cell lines were cultured in Dulbecco’s modified Eagle’s medium (DMEM); human colon cancer cell line HCT116 was cultured in McCoy’s 5A medium (Invitrogen). Both media were supplemented with 10% fetal bovine serum (FBS) and cells were maintained under 5% CO_2_ at 37 °C. The vector control and two stable DDX3-knockdown HuH7 cell lines, siDDX3-433-33 and siDDX3-433-52 (kindly provided by Dr. Pei-Ching Chang, National Yang-Ming University), were maintained in above DMEM culture media with 200 μg/ml hygromycin B (Calbiochem). Cell transient transfections were performed using either calcium phosphate co-precipitation method or by Lipofectamine 2000 transfection reagent (Invitrogen), the later was done according to the manufacturer’s instructions.

### HCV preparation and virus infection assay

J6/JFH virus was produced and titrated as described previously^69^. Huh-7.5.1 cells were seeded one day before infection. On the day of infection, cells were incubated with J6/JFH virus at an multiplicity of infection (MOI) of 0.5 for 6 hr and then washed twice with fresh medium. Two days later, cells were collected, lysed and subjected to western blotting.

### Enzyme-linked immunosorbent assay of secreted ApoB

Culture media were collected after centrifugation at 3,000× *g* for 10 minutes to remove debris. Culture media and ApoB standard were subjected to the assay with Human Apolipoprotein B ELISA Kit (Abcam) following manufacturer’s instruction.

### Detection of intracellular triglyceride

Detection of intracellular triglyceride was performed using Triglycerides FS and its associated standard purchased from Diagnostic Systems. The assay was performed according to manufacturer’s instruction. In brief, 5 × 10^5^ cells of parental HuH7 as well as the vector control and two stable DDX3-knockdown HuH7 cell lines, siDDX3-433-33 and siDDX3-433-52 cells were seeded into 6-well plate. After 48 hr, cells were harvested and lysed by RIPA buffer (Bio Basic Canada Inc.). After centrifugation at 12,000 r.p.m. at 4 °C for 30 minutes, supernatants were collected. Supernatant and standard solution were incubated separately with assay reagent at room temperature for 20 minutes. Absorbance at 500 nm wavelength was measured by spectrometer and converted to absolute concentration following manufacture’s protocol.

### Plasmid constructions and purification of recombinant GST fusion proteins

pFL-J6/JFH plasmid^70^ encoding the full-length J6/JFH HCV genome was used to prepare J6/JFH virus stock (genotype 2a) and was kindly provided by Dr. Charles M. Rice (Rockefeller University). The mammalian expression plasmids pcDNA/HA-DDX3, pcDNA/HA-DDX3(DQAD) and pcDNA/HA-DDX3(AAA) were constructed by subcloning of the *Eco* RI/*Apa* I-digested DNA fragments PCR amplified from plasmids pcDNA-SRα/Fag-DDX3, pcDNA-SRα/Fag-DDX3(DQAD) and pcDNA-SRα/Fag-DDX3(AAA)^23^ into *Eco* RI/*Apa* I-treated pGL2-basic vector (Promega Biotech), respectively. The pEGFP-DDX3 plasmid has been described previously^20^. To construct the mammalian expression plasmids pcDNA/HA-SHP and pcDNA/Flag-SHP, the *Eco* RI/*Xba* I-digested DNA fragment of *SHP* gene derived from RT-PCR were inserted into *Eco* RI/*Xba* I-treated pcDNA/HA and pcDNA/Flag vectors, respectively. The plasmids pcDNA/HA-HNF4 and pcDNA/Flag-HNF4 were constructed by subcloning the *Eco* RI/*Apa* I-digested DNA fragment containing *HNF4* gene derived from RT-PCR into *Eco* RI/*Apa* I-treated pcDNA/HA and pcDNA/Flag vector, respectively. The reporter plasmid pGL2/MTP-Luc(−611/+87) harbored *MTP* promoter region (from nucleotides −611 to +87 relative to the transcription start site, harboring two HNF4 responsive elements) was constructed by subcloning the promoter fragment PCR amplified from HuH7 genomic DNA into the *Sma* I site of pGL2-basic vector. The bacteria expression plasmids pGEX-HNF4/1-127, pGEX-HNF4/128–370 and pGEX-HNF4/371–465, which express the GST-tagged HNF4 derivatives, were constructed by insertion of PCR-generated HNF4 DNA fragments encoding amino acids 1–127, 128–370 and 371–465 residues into *Eco* RI/*Not* I-digested pGEX-5X-1 vector (GE Healthcare). The pGEX-5X-1/DDX3 was used to express GST-tagged full length DDX3^21^. pMTP(mutant)-Luc reporter plasmid in which both the HNF4-responsive elements were mutated were constructed by site-directed mutagenesis of pGL2/MTP-Luc(−611/+87) using the QuikChange site-directed mutagenesis system (Stratagene) with primers: 5′-GTG AGC CCT TCA GTG TTG TTA CCT CCT GAT TTT GGA GTT TGG AGT CTG TGC TTT CCC C-3′ and 5′-GGG GAA AGC ACA GAC TCC AAA CTC CAA AAT CAG GAG GTA ACA ACA CTG AAG GGC TCA C-3′. The integrity of above-mentioned plasmids was verified by nucleotide sequencing. The siRNAs (siControl and siDDX3) were purchased from Thermo Scientific Dharmacon RNAi Technologies. The *E. coli* BL21 (DE3) expressed recombinant GST-fusion proteins were amplified and purified as described previously^18^.

### Oil Red O staining

Oil Red O staining was carried out as described previously^71^ with minor modification. Briefly, HuH7 and HepG2 cells were transient transfected with 33 nM siRNA (siControl and siDDX3) using Lipofectamine 2000 (Invitrogen), respectively. After 72 hr, cells were washed twice with PBS and fixed with 10% formaldehyde for 30 min at RT. The fixed cells were then incubated with 60% isopropanol for 5 min to eliminate background staining, followed by incubation freshly prepared Oil Red O working solution (0.3% Oil Red O in 60% isopropanol) at RT for 1 hr. After several rinses with distilled water, the images were observed by light microscopy. Absorbance (500 nm) of 100% isopropanol eluted Oil Red O was then detected by a spectrophotometer. Similarly, the measurement of intracellular neutral triglycerides and lipids in two DDX3 knockdown cell lines (siDDX3-433-33 and siDDX3-433-52) and parental HuH7 cells were also performed.

### *In vitro* GST pull-down assay

The bacteria expression plasmids pGEX-HNF4/1–127, pGEX-HNF4/128–370 and pGEX-HNF4/371–465, which produce the GST-tagged HNF4 derivatives, were transformed and expressed in *E. coli* BL21 (DE3). The GST pull down assay was conducted in accordance with the procedure described previously^18^. In brief, 20 μl glutathione Sepharose resins (GE Healthcare) prebound GST fusion proteins (3 μg) were incubated with nuclear extracts (200 μg, prepared as described previously^18^) of HuH7 or HepG2 cells expressing either HA-tagged SHP or HA-tagged HNF4 in the presence or absence of 100 μg/ml RNAse A. After incubation at 4 °C for 5 hr, the beads were washed thrice with cold PBS containing 0.5% NP40. The associated proteins were eluted and separated by SDS-PAGE, then subjected to Western blot analysis with anti-HA antibody (Roche).

### *In vivo* co-immunoprecipitation

HuH7 cells were seeded on 100 mm dishes to approximately 60–70% confluence. After overnight incubation, cells were transfected with appropriate amount of expression plasmids using calcium phosphate coprecipitation method. Forty-eight hours later, nuclear extracts were prepared and co-IP was performed as described previously^18^. Briefly, nuclear extracts (200 μg) were incubated with 20 μl anti-HA agarose beads (Sigma-Aldrich), anti-Flag M2 agarose resins (Sigma-Aldrich) or protein G Sepharose-conjugated rabbit anti-p300 (Santa Cruz) and anti-CBP (Santa Cruz) in co-IP buffer (20 mM Tris-HCl pH 7.5, 150 mM NaCl, 10% glycerol, 1% Triton X-100 and protease inhibitor) at 4 °C overnight. The beads with immunoprecipitates were rinsed thrice with cold PBS containing 0.5% NP40. The eluted complexes were subjected to SDS-PAGE followed by immunoblotting with antibodies against HA and Flag (Sigma-Aldrich).

### RNA isolation and quantitative real-time RT-PCR

Total RNA was extracted from HuH7 cells by using Tri reagent (Sigma-Aldrich) and cDNA was synthesized using 2 μg of total RNA with Superscript II reverse transcriptase (Invitrogen) and oligo dT as primer. These two experiments were conducted in accordance with the manufacturer’s instructions. The quantitative PCR was performed with SYBR Green I LightCycler system (Roche) as described previously^24^. The primer set sequences for amplification of *DDX3* are 5′-GAAGCTACTAAAGGTTTCTAC-3′ (forward) and 5′-TCTCAACATCACTGAAACTTTC-3′ (reverse); primer set sequences for *MTP* are 5′-GGAGCTTCCCCAAGAAATGAAT-3′ (forward) and 5′-GAACTCGACGGACAATTTCT-3′ (reverse); primer set sequences for internal control *GAPDH* are 5′-CAACTACATGGTTTACATGTTC-3′ (forward) and 5′-GCCAGTGGACTCCACGAC-3′ (reverse).

### Luciferase assay

Approximately 60–70% confluent HuH7, HepG2, HCT116 and HeLa cells in 6-well plates were transfected with pGL2/MTP-Luc(−611/+87) or pMTP(mutant)-Luc reporter plasmid alone or together with various amounts of HA-HNF4, HA-SHP and HA-DDX3 expression constructs (as indicated in the figure legends) using lipofectamine 2000 transfection reagent or calcium phosphate coprecipitation method. Forty-eight hr posttransfection, cells were harvested and subjected to luciferase assay either as described previously^18^ or using the Luciferase Assay System (Promega) according to the manufacturer’s instruction.

### *In vitro* biotinylated probe binding assay

The WT (5′-CAGTGAACTTAGGTCCTGATT-3′) and mutant form (5′-CAGTGTTGTTAGGTCCTGATT-3′) of the 5′ biotinylated probes containing HNF4 responsive element were annealed with their complementary oligonucleotides in 20 μl of annealing buffer (40 mM Tris-HCl pH7.5, 20 mM MgCl_2_ and 50 mM NaCl) at 80 °C for 5 min. Nuclear extracts (300 μg) of transfected HuH7 cells were precleared with streptavidin-agarose resins (Sigma) and then incubated with 125 mg/ml poly(dI.dC) (Sigma) at 4 °C for 30 min in 300 μl binding buffer (18 mM HEPES pH 7.9, 40 mM KCl, 2 mM MgCl_2_, 10 mM DTT and protease inhibitor). The mixtures were incubated with annealed biotinylated probe and 20 μl of streptavidin-agarose resins at room temperature for 1 hr. After extensive wash with binding buffer, the associated protein complexes were analyzed by SDS-PAGE, followed by immunoblotting with anti-HA antibody.

### Chromatin immunoprecipitation (ChIP) assay

ChIP assay was performed as described previously with minor modification^72^. In brief, HuH7 cells were cotransfected with construct encoding Flag-HNF4 alone or together with HA-DDX3 expression plasmid. After 48 hr, cells were fixed with 1% formaldehyde and incubated at 37 °C for 10 min, then glycine was added to a final concentration of 0.125M at room temperature for 5 min to stop the cross-link reaction. Cells were harvested and lysed using SDS-lysis buffer (50 mM Tris-HCl pH 8.1, 100 mM NaCl, 0.5% SDS, 5mM EDTA and protease inhibitor) to isolate nuclei. The nuclei were resuspended in IP buffer (100 mM Tris-HCl pH 8.6, 0.3% SDS, 1% Triton X-100, 5 mM EDTA and protease inhibitor) and the nuclear extracts were sheared by sonication to average length of DNA fragments about 500 bp. The chromatin complexes were immunoprecipitated with anti-Flag M2 resins at 4 °C overnight. After extensive wash, immunoprecipitates were eluted and the immunoprecipitated DNA was isolated by reversing the cross-link using the Qiaquick PCR purification kit (Qiagen). The purified DNA fragments were detected by PCR amplication with *MTP* promoter-specific primers. The primer set sequences for *MTP* promoter are: 5′-CTGGTTTGGTTTAGCTCTC-3′ (forward) and 5′-GACCCTCTTCAGAACCTG-3′ (reverse).

### *In situ* proximity ligation assay (PLA)

Plasmid-transfected HuH7 cells were fixed with 4% paraformaldehyde 48 hr post-transfection. Fixed cells were permeabilized with 0.1% Triton X-100, blocked with 3% BSA, incubated with primary antibodies diluted in 1% BSA and then with either anti-mouse MINUS or anti-rabbit PLUS PLA probes and subjected to the ligation and amplification reactions using the Duolink *in situ* kit (Sigma-Aldrich). Images were acquired with the Zeiss LSM 700 confocal microscope. Number of PLA signals were quantified using the MetaMorph software (Molecular Devices).

## Additional Information

**How to cite this article**: Tsai, T.-Y. *et al*. RNA helicase DDX3 maintains lipid homeostasis through upregulation of the microsomal triglyceride transfer protein by interacting with HNF4 and SHP. *Sci. Rep.* **7**, 41452; doi: 10.1038/srep41452 (2017).

**Publisher's note:** Springer Nature remains neutral with regard to jurisdictional claims in published maps and institutional affiliations.

## Supplementary Material

Supplementary Figures
