# Supplementary material for: RNA helicase DDX3 maintains lipid homeostasis through upregulation of the microsomal triglyceride transfer protein by interacting with HNF4 and SHP
Source: Sci Rep. 2017 Jan 27;7:41452. doi: 10.1038/srep41452 (PMC5269733; doi:10.1038/srep41452)
Supplement: Supplementary Figures [file srep41452-s1.pdf]

Scientific Reports
Supplementary information

RNA helicase DDX3 maintains lipid homeostasis through upregulation of the microsomal triglyceride transfer protein by interacting with HNF4 and SHP

Tsung-Yuan Tsai, Wei-Ting Wang, Hao-Kang Li, Wei-Ju Chen, Yu-Hong Tsai, Chi-Hong Chao and Yan-Hwa Wu Lee

Supplementary Figure S1 and S2

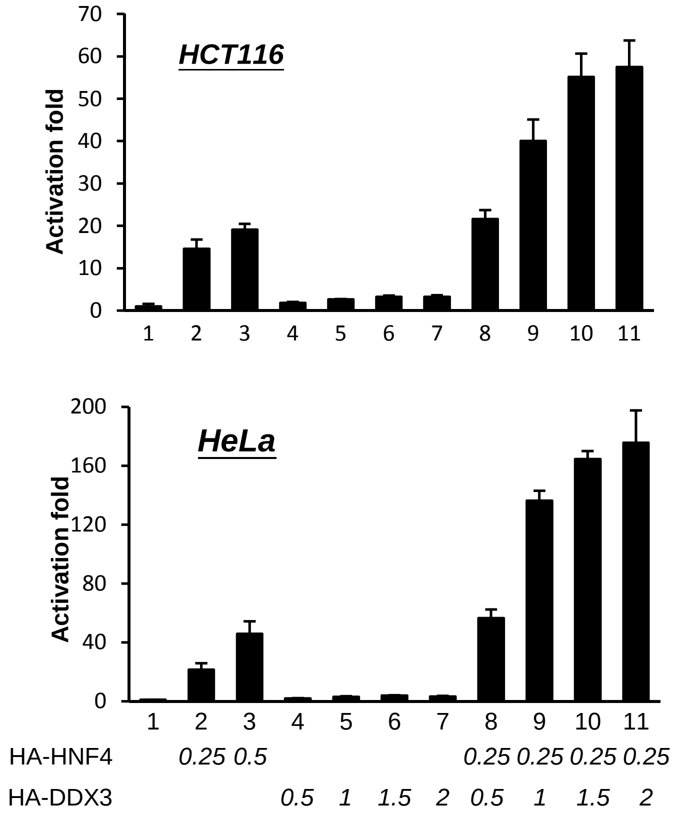

Supplementary Figure S1. DDX3 up-regulates the HNF4-mediated transactivation of MTP promoter in HCT116 and HeLa cells

MTP promoter (-611 ~ +87) -driven reporter plasmid (pGL2/MTP-Luc(-611/+87) , 0.25 μg) was transfected alone or together with HA-HNF4 and HA-DDX3 expressing constructs as indicated amount in HCT116 and HeLa cells by lipofectamine 2000. The total amount of transfected plasmids was adjusted to 2.5 μg/35 mm dish by supplementing with control vector, pcDNA/HA. Forty-eight hours later, cells were collected and subjected to luciferase activity assay.

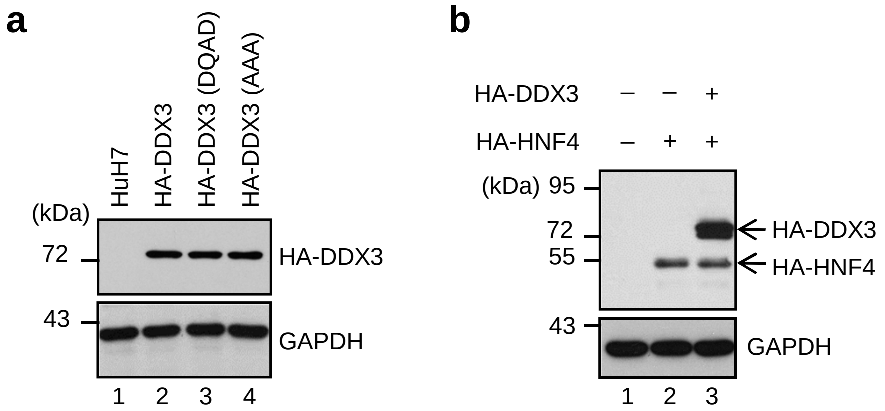

Supplementary Figure S2. Similar protein expression levels among wild type and mutant DDX3 and the exogenous HNF4 protein levels with DDX3 cotransfection
a. The exogenous expression levels of wild type and mutant types of HA-DDX3. HuH7 cells were transfected with a fixed amount of expressing constructs for HA-DDX3(WT), HA-DDX3(DQAD) and HA-DDX3(AAA). Total cell lysates (20 μg) for each transfected cells were subjected to SDS-PAGE and followed by Western blot analysis. GAPDH was served as a loading control. **b.** The expression level of HA-HNF4 was not affected by adding HA-DDX3 in HuH7 cells. Equal volumes of cell lysates from transfected HuH7 cells (Supplementary Figure S1 panel b: lane 1, lane 2 and lane 11) were subjected to SDS-PAGE and immunoblotting with anti-HA antibody.
